# Tumor Necrosis Factor Alpha: A Link between Neuroinflammation and Excitotoxicity

**DOI:** 10.1155/2014/861231

**Published:** 2014-05-21

**Authors:** Gabriel Olmos, Jerònia Lladó

**Affiliations:** Grup de Neurobiologia Cel**·**lular, Departament de Biologia and Institut Universitari d'Investigacions en Ciències de la Salut, IUNICS, Universitat de les Illes Balears, 07122 Palma de Mallorca, Spain

## Abstract

Tumor necrosis factor alpha (TNF-**α**) is a proinflammatory cytokine that exerts both homeostatic and pathophysiological roles in the central nervous system. In pathological conditions, microglia release large amounts of TNF-**α**; this *de novo* production of TNF-**α** is an important component of the so-called neuroinflammatory response that is associated with several neurological disorders. In addition, TNF-**α** can potentiate glutamate-mediated cytotoxicity by two complementary mechanisms: indirectly, by inhibiting glutamate transport on astrocytes, and directly, by rapidly triggering the surface expression of Ca^+2^ permeable-AMPA receptors and NMDA receptors, while decreasing inhibitory GABA_A_ receptors on neurons. Thus, the net effect of TNF-**α** is to alter the balance of excitation and inhibition resulting in a higher synaptic excitatory/inhibitory ratio. This review summarizes the current knowledge of the cellular and molecular mechanisms by which TNF-**α** links the neuroinflammatory and excitotoxic processes that occur in several neurodegenerative diseases, but with a special emphasis on amyotrophic lateral sclerosis (ALS). As microglial activation and upregulation of TNF-**α** expression is a common feature of several CNS diseases, as well as chronic opioid exposure and neuropathic pain, modulating TNF-**α** signaling may represent a valuable target for intervention.

## 1. Introduction

Tumor necrosis factor alpha (TNF-*α*) was originally identified as a factor that leads to rapid necrosis of transplantable tumors in mice [1] and now it is considered a proinflammatory cytokine involved in the innate immune response [2]. In the central nervous system (CNS) TNF-*α* exerts both homeostatic and pathophysiological roles [3, 4]. In the healthy CNS TNF-*α* has regulatory functions on crucial physiological processes such as synaptic plasticity [5, 6], learning and memory [7, 8], sleep [9], food and water intake [10], and astrocyte-induced synaptic strengthening [11]. In pathological conditions, astrocytes and mainly microglia release large amounts of TNF-*α*; this* de novo* production of this cytokine is an important component of the so-called neuroinflammatory response that is associated with several neurological disorders [3, 12–14]. In addition, TNF-*α* can potentiate glutamate-mediated cytotoxicity by two complementary mechanisms: indirectly, by inhibiting glutamate transport on astrocytes, and directly, by increasing the localization of ionotropic glutamate receptors to synapses [15]. Neuroinflammation and excitotoxicity have key roles as triggers and sustainers of the neurodegenerative process and thus, elevated levels of TNF-*α* have been found in traumatic brain injury [16], ischemia [17, 18], Alzheimer's disease (AD) [19, 20], Parkinson's disease (PD) [21, 22], multiple sclerosis (MS) [23, 24], and amyotrophic lateral sclerosis (ALS) [25, 26]. This review summarizes the current knowledge of the cellular and molecular mechanisms by which TNF-*α* potentiates excitotoxicity and describes its key role in linking the neuroinflammatory and excitotoxic processes that take place not only in ALS but also in other common neurodegenerative diseases.

## 2. TNF-*****α***** Signaling

TNF-*α* is first synthesized as a transmembrane protein (tmTNF-*α*). The cleavage of the extracellular domain of tmTNF-*α* by the matrix metalloprotease TNF-*α*-converting enzyme (TACE) releases a soluble TNF-*α* (sTNF-*α*) homotrimer. Remarkably, both tmTNF-*α* and sTNF-*α* are biologically active and their signal transduction involves binding to two distinct surface receptors, TNF-*α* receptor 1 (TNFR1 or p55TNFR) and TNF-*α* receptor 2 (TNFR2 or p75TNFR), which are different in their expression pattern, downstream signal-transduction cascades, and binding affinity for TNF-*α* [27–29]. The cytoplasmic tail of TNFR1 contains a death domain; however, this motif is missing in TNFR2. Although initially it was considered that TNFR1 activation was involved in the cytotoxic and apoptotic effects of TNF-*α*, and those related to cell survival and proliferation involved TNFR2 activation, now it is becoming clear that TNFR2 can also induce cell death [30]. Binding of the TNF-*α* trimer to the extracellular domain of TNFR1 induces receptor trimerization and recruitment of the adaptor protein TNF receptor-associated death domain (TRADD), which in turn recruits additional adaptor proteins: receptor-interacting protein (RIP), TNF receptor-associated factor 2 (TRAF2), and Fas-associated death domain (FADD). This latter protein mediates recruitment and activation of caspases 8 and 10 that initiate a protease cascade that leads to apoptosis [31]. TNFR1 signaling also results in the activation of the following signal transduction pathways: the nuclear factor-kappa B (NF-*κ*B), the extracellular signal-regulated kinase (ERK), the c-Jun N-terminal kinase (JNK), the p38 mitogen-activated protein kinase (p38 MAPK), the acidic sphingomyelinase (A-SMase), and the neutral sphingomyelinase (N-SMase) pathways. These pathways regulate the expression of several genes, and some of them, particularly those regulated by the NF-*κ*B pathway, have antiapoptotic effects. With the exception of the SMase pathways, the above signal transduction pathways can also be induced through TNFR2 signaling because TRAF2 (along with TRAF1) can directly associate to the intracellular domain of the TNFR2 receptor (reviewed by [32, 33]).

## 3. TNF-*****α***** and Neuroinflammation

Neuroinflammation in the CNS refers to the collective response of microglia, and to a lesser extent of astrocytes and oligodendrocytes, against diverse insults (invading pathogens, trauma, aggregated or modified proteins, stroke, etc.) designed to remove or inactivate the noxious agents and to inhibit and reverse their detrimental effects. The glial response can be considered as an innate immune mechanism, whereas the participation in the neuroinflammatory process of lymphocytes (mainly T cells) carrying binding sites for specific antigens is an acquired immune mechanism [14]. In neurodegenerative diseases, both innate and acquired immune mechanisms are unable to resolve the triggers, creating a self-sustaining environment where the neuroinflammation persists, thus leading to a chronic neuroinflammation.

**Figure 1:**
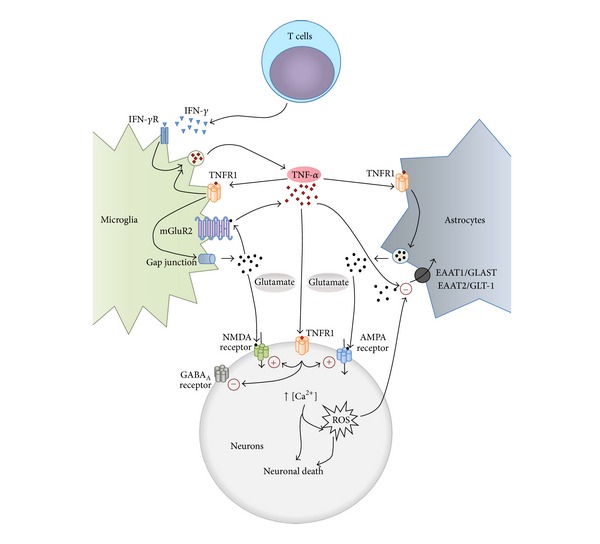
Proposed mechanisms by which TNF-*α* links the neuroinflammatory and the excitotoxic processes. The cytokine IFN-*γ*, released by infiltrated T cells, activates TNF-*α* production and release in microglia. TNF-*α*, through TNFR1 signaling, promotes further microglial TNF-*α* release and also induces glutamate release from hemichannels of gap junctions. In astrocytes, TNF-*α* stimulates TNFR1 to induce glutamate exocytosis and also inhibits glutamate uptake, thus increasing extracellular glutamate levels. In neurons TNF-*α*, via TNFR1, rapidly increases the excitatory synaptic strength by inducing increased Ca^2+^ permeable-AMPA receptors and/or NMDA receptors and also decreases the surface expression of inhibitory GABA_A_ receptors. The excessive Ca^2+^ input to neurons induces neuronal death and generates excessive ROS that disrupt glutamate transport in neighboring astrocytes. The dying neurons maintain microglia in an active state, releasing TNF-*α*.

Although astrocytes and neurons are able to produce TNF-*α* [34–36], it is assumed that microglia are the major source of this cytokine during neuroinflammation [37, 38]. The cytokine interferon gamma (IFN-*γ*) is a potent inducer of TNF-*α* gene expression in microglia [38–40], and also it upregulates the expression of adhesion/costimulatory molecules, like the major histocompatibility complex (MHC) class II molecules to sustain antigen-dependent T-cell activation [41, 42]. The different inflammatory stimuli that activate microglia during neuroinflammation trigger different signaling pathways including p38 MAPK, JNK, NF-*κ*B, and ERK1/2 [43–46], making it difficult to determine which of them is in fact implicated in the induction of TNF-*α* expression. In our laboratory, we demonstrated that the sole inhibition of the mitogen-activated protein kinase and ERK kinase (MEK)/ERK signaling pathway with U0126 or apigenin was enough to inhibit the LPS or the IFN-*γ*-stimulated TNF-*α* expression in the BV-2 microglial cell line [47]. Similar results had been previously published in human monocytes [48]. As IFN-*γ* is produced by T cells but not in significant amounts by any CNS resident cells, including microglia [49, 50], it has been proposed that, in neurodegenerative diseases, activated T cells would infiltrate into the parenchyma of the CNS [51–54] releasing their own inflammatory mediators, including IFN-*γ* [55, 56]. In this sense, in ALS, lymphocytic infiltrates and antibody deposits were detected in postmortem CNS tissues [57] and, more recently, increased CD4^+^ and CD8^+^ T cells were found to invade the brain in postmortem human specimens and in a mouse model of PD [58]. Although the role of the infiltrated T cells in the CNS is controversial, since both CD4^+^ and CD8^+^ T cells can have detrimental [59] or protective effects [60–62], it may be proposed that, during the neuroinflammatory process, these infiltrated cells release the cytokine IFN-*γ* which, via the MEK/ERK signaling pathway, induces in microglia an increased* de novo* TNF-*α* production and release (Figure 1). However, T cells may not be present in the CNS at early disease stages; for example, in ALS, T cells infiltrate the spinal cord as disease progresses [63]. Thus, microglia can be activated to release TNF-*α* at early asymptomatic disease stages by sensing the earliest neuronal stress and later, infiltrated T cells releasing IFN-*γ* would keep microglia in an active state [64, 65].

These findings indicate that IFN-*γ* and TNF-*α* have key roles in the glial-T-cell dialogue that occurs during neuroinflammation, as they are involved in T-cell adhesion to endothelial cells, extravasation, and T-cell and glial activation [54]. In this regard, we have demonstrated that IFN-*γ* and TNF-*α* have complementary roles in inducible microglial nitric oxide generation [47] and that both cytokines, through the induction of the expression of several prooxidative enzymes, cooperatively induce oxidative stress and motoneuron death [66].

## 4. TNF-*****α***** and Excitotoxicity

Glutamate is the main excitatory neurotransmitter in the mammalian CNS and is involved in many aspects of normal brain function [67]. Excitotoxicity refers to a process of neuronal death caused by excessive or prolonged activation of receptors for this excitatory amino acid [68]. A role for excitotoxicity in the aetiology or progression of many human acute or chronic neurodegenerative diseases, including ischemia, AD, PD, MS, and ALS has been proposed (reviewed by [69]).

The first reports demonstrating that TNF-*α* was able to potentiate excitotoxicity were performed in human neuronal cultures. Gelbard et al. demonstrated that subtoxic doses of both TNF-*α* and *α*-amino-3-hydroxy-5-methyl-4-isoxazolepropionic acid (AMPA) were neurotoxic when combined [70]. Similar results were published one year later showing that TNF-*α* potentiated glutamate neurotoxicity and that this effect could be blocked by competitive (2-APV) and noncompetitive (MK-801) NMDA receptor antagonists [71]. Later, and by working on rat spinal cord, it was demonstrated that nanoinjections of nontoxic doses of either TNF-*α* or kainate (KA) alone into the thoracic gray matter resulted in almost no tissue damage; however, the combination of these substances at the same doses produced a large area of tissue necrosis and neuronal cell death, an effect that could be reverted by the AMPA receptor antagonist 6-cyano-7-nitroquinoxaline 2,3-(1*H*,4*H*)-dione (CNQX) [72].

## 5. Potentiation of Excitotoxicity by TNF-*****α*****: Role of Glial Cells

After the above previous works, the role of glial cells in TNF-*α* induced neuronal death was investigated. In mouse primary microglia it was demonstrated that TNF-*α*, through the TNFR1 pathway, induces excitotoxicity by promoting microglial glutamate release from hemichannels of gap junctions in an autocrine manner [73]. Moreover, in rat primary microglia it was found that agonist stimulation of group 2 metabotropic glutamate receptors (mGluR2) induced TNF-*α* release, and when this microglial-conditioned medium was added to cerebellar granule neurons in culture resulted in caspase-3 activation and apoptotic cell death. The authors also identified microglial-released Fas ligand as an essential cofactor for the TNF-*α*-induced neurotoxicity [74]. Similar results were obtained on hippocampal neurons where TNF-*α* derived from KA-activated microglia also resulted in apoptotic neuronal cell death [75]. Thus, two potential microglial autocrine loops participating in excitotoxicity can be identified: first, TNF-*α* promotes further microglial TNF-*α* production and release through TNFR1 signaling [76] and second, TNF-*α* induces glutamate release that acts on microglial mGluR2 to induce more TNF-*α* production (Figure 1).

In astroglia, the interaction of TNF-*α* with TNFR1 initiates a sequence of intracellular signaling events that leads to generation of prostaglandin E_2_ that, in turn, activates the elevation of intracellular calcium followed by glutamate exocytosis [77, 78]. The excessive glutamate concentrations resulting from TNF-*α* stimulation of astroglial and microglial TNFR1 could be diminished by the glutamate uptake system [79, 80]; however, TNF-*α* has a detrimental effect on astroglial glutamate uptake (reviewed by [81]) (Figure 1). At least five sodium-dependent glutamate transporters have been cloned. The transporters (human/rat) EAAT1/GLAST and EAAT2/GLT-1 are predominantly located on astrocytes and GLT-1 is the most abundant glutamate transporter in the adult brain [82, 83]. In human H4 astroglioma cells and rat astrocytes, it has been shown that exposure for four to six hours to TNF-*α* (10 or 20 ng/mL) inhibits glutamate uptake by inducing a downregulation of EAAT2/GLT-1 mRNA [84, 85]. In H4 astroglioma cells, downregulation of EAAT2 was dependent on the TNF-*α* induced binding of NF-*κ*B to the EAAT2 promoter [84]. The role of NF-*κ*B in regulating GLT-1 expression was further confirmed in our laboratory. We used spinal cord organotypic cultures to create a model of chronic glutamate excitotoxicity in which glutamate transporters were inhibited by threohydroxyaspartate (THA) to induce motoneuron death. Exposure to THA induced microglial activation and TNF-*α* release. In the presence of exogenous TNF-*α* (20 ng/mL), THA-induced excitotoxic motoneuron death was potentiated. Coexposure to TNF-*α* and THA also resulted in downregulation of GLT-1 and in increased extracellular glutamate levels. The downregulation of GLT-1, as well as the excitotoxic motoneuron death, could be prevented by NF-*κ*B inhibition [86].

When TNF-*α* (20 ng/mL) was applied for a short time period (30 minutes) in hippocampal-entorhinal complex slice cultures, it reduced glutamate transport without affecting GLT-1 or GLAST expression [87]. The mechanisms of this rapid, and apparently, nongenomic effect of TNF-*α* are not clear. On the one hand, TNF-*α* is a clear inducer of oxidative stress in the CNS [66, 88, 89], and evidences indicate that glutamate transporters are vulnerable to the action of reactive oxygen and nitrogen species that inhibit glutamate uptake within minutes [90, 91], thus providing a link between oxidative stress and excitotoxicity. In addition, reactive oxygen species generated within neurons in response to an excitotoxic insult can pass across the plasma membrane and disrupt glutamate transport in neighboring astrocytes [92]. On the other hand, TNF-*α*, as explained before, can activate caspases, including caspase-3, which can also be activated by oxidative stress [93]. In this sense, caspase-3 mediated cleavage of GLT-1 results in the inhibition of its activity [94].

It is interesting to note that, in those neurological disorders in which neuroinflammation and increased levels of TNF-*α* have been described (see Section 1), it has also been reported a reduced expression of GLT-1, GLAST, or both (reviewed by [81]). As an example, in ALS, where neuroinflammation and excitotoxicity are fundamental mechanisms involved in motoneuron degeneration [65, 95], decreased GLT-1 expression has been reported both in patients [96] and rat models [97, 98]. Moreover, the intrathecal injection of cerebrospinal fluid from ALS patients in the rat spinal cord or the* in vitro* exposure to this fluid of mixed spinal cord cultures also resulted in a decrease of GLT-1 expression [99].

## 6. Potentiation of Excitotoxicity by TNF-*****α*****: Modulation of Glutamate and GABA_**A**_ Receptors

AMPA-type glutamate receptors (AMPARs) are ligand-gated channels that mediate fast excitatory synaptic transmission in the vertebrate CNS. These receptors are tetramers assembled from glutamate receptor (GluR) 1, 2, 3, and 4 (or GluR-A, -B, -C, and -D) subunits around an aqueous pore in the membrane [100, 101]. The trafficking of AMPARs with different subunit composition was initially described in hippocampal CA1 pyramidal cells [102], and now it is generally accepted that such trafficking is critical for the modulation of synaptic strength during learning and memory. Thus, AMPARs trafficking has been implicated in homeostatic synaptic scaling and other forms of long-term synaptic strength adjustments [4, 103, 104]. The GluR2 subunit has a key role in determining the permeability to Ca^2+^ of AMPARs. GluR2 in combination with other GluR subunits forms channels that are Ca^2+^-impermeable [105, 106]. In this regard, in 1997 it was proposed the “GluR2 hypothesis” suggesting that the selective vulnerability of specific neuron populations, described in some neurological disorders, was due to a reduction in the AMPARs expressing the GluR2 subunits, resulting in increased density of Ca^2+^-permeable AMPARs. The consequent increase in Ca^2+^ influx through these channels would result in a loss of Ca^2+^ homeostasis, thus contributing to the delayed neurodegeneration seen in those conditions [107]; see also [108].

TNF-*α* has an important role in the regulation of AMPARs trafficking being a critical component of the homeostatic regulatory system controlling synaptic plasticity [4]. In 2002, Yu et al. demonstrated that human NT2-N neurons exposed to TNF-*α* increased their expression of the GluR1 subunit, resulting in an increased susceptibility to KA-induced necrosis. The effect of TNF-*α* implicated both the A-SMase and the NF-*κ*B signaling pathways [109]. Similar results were obtained in hippocampal neurons where TNF-*α*, within 15 min, increased the surface expression of GluR1-containing AMPARs, and these changes were accompanied by dramatic changes in AMPAR-mediated excitatory postsynaptic currents [5]. Later, it was demonstrated a dual role for TNF-*α* on AMPA-induced excitotoxicity. In mouse hippocampal slice cultures it was reported that pretreatment (24 h) of cultures with 10 ng/mL TNF-*α* potentiated AMPA-induced neuronal death; however, decreasing the concentration of TNF-*α* to 1 ng/mL resulted in neuroprotection. The authors demonstrated that the “high-dose” toxic effect was mediated by TNFR1 whereas the “low-dose” protective effect implied the TNFR2 [110]. The role of TNFR1 in the potentiation by TNF-*α* of AMPA toxicity was further confirmed in a work performed also in hippocampal cells and demonstrating that, by activating neuronal TNFR1, TNF-*α* increased the surface AMPARs, but remarkably, TNF-*α* preferentially increased the synaptic expression of GluR2-lacking (Ca^2+^ permeable) AMPARs. This effect was mediated through a phosphatidylinositol 3-kinase- (PI3-K-) dependent process [111]. The role of PI3-K in the potentiation by TNF-*α* of KA-induced neuronal death was confirmed later by the same group; they demonstrated that the specific PI3-K inhibitor LY294002 reverted the TNF-*α* effect on hippocampal neurons. Moreover, and in agreement with the “GluR2 hypothesis,” the potentiating effect was also reverted by the synthetic joro spider toxin analog NASPM, which selectively blocks Ca^2+^ permeable-AMPARs [112]. These results were also confirmed by others showing that TNF-*α* triggers a rapid induction of Ca^2+^ permeable-AMPARs in hippocampal pyramidal neurons; the effect was rapid (15 min) and since TNF-*α* exposure did not alter mRNA levels for either GluR1 or GluR2 subunit, it was proposed that TNF-*α* acts at posttranscriptional level to induce rapid increases in surface subunit expression [113].

The pathological relevance of the above findings was first described in a model of cervical spinal cord contusion injury. In this model, increased synaptic AMPAR numbers were found at synapses ipsilateral to the lesion at 90 min and 3 h after injury. Interestingly,* in vivo* nanoinjections of TNF-*α* into the ventral horns resulted in increased GluR1 and decreased GluR2 at both extrasynaptic and synaptic plasma membrane sites. The effect was seen in the neuropil 60 min after TNF-*α* nanoinjection and could also be detected in the somata of large spinal motoneurons [114]. In a subsequent study, using whole cell recording from lumbar motoneurons, it was demonstrated that both AMPA and NMDA receptor-mediated excitatory postsynaptic currents were rapidly increased following bath application of TNF-*α* [115]. Together, these results suggested that TNF-*α* induced GluR2-lacking AMPARs trafficking to the membrane is likely to contribute to postinjury excitotoxicity in spinal cord motoneurons. However, another study has reported TNF-*α* to reduce AMPAR-mediated calcium entry in cultured motoneurons by increasing cell surface expression of the GluR2 subunit [116].

Adult spinal cord motoneurons possess significant numbers of Ca^2+^ permeable-AMPARs under basal conditions, and it has been proposed that this circumstance would render them more susceptible to neurodegeneration in ALS [117–120]. Activation of both microglia and astrocytes occurs prominently in both human disease and animal models of ALS [121, 122]; these activated cells may contribute to motoneuron injury by releasing TNF-*α* [89, 123]. In this sense, TNF-*α* has been shown to potentiate AMPAR-mediated excitotoxicity on lumbar spinal cord motoneurons both by decreasing GLT-1 expression [86], and also by inducing a rapid membrane insertion of Ca^2+^ permeable-AMPARs via a PI3-K and protein kinase A- (PKA-) dependent mechanism [124]. Interestingly, vascular endothelial growth factor (VEGF) has neuroprotective effects on ALS (reviewed by [125]), and it has been shown, both* in vitro* and* in vivo*, that VEGF increases the expression of GluR2 subunit of AMPARs of spinal cord motoneurons, thus minimizing their vulnerability to AMPA-mediated excitotoxicity [126].

The effects of TNF-*α* on N-methyl-D-aspartate receptors (NMDARs) trafficking are less studied; however, the results obtained are similar to those on AMPARs. Thus, in hippocampal neurons TNF-*α* induced a rapid increase in the surface expression of the NR1 subunit of NMDARs and also, via N-SMase2, promoted a specific clustering of phosphorylated NR1 subunits into lipid rafts [127]. Similarly to that described above for motoneurons, TNF-*α* has also been shown to potentiate NMDAR-mediated excitotoxicity in cortical neurons [128].

TNF-*α* also regulates inhibitory synapse function. An* in vivo* study in the rat spinal cord indicated that TNF-*α* increased within 60 min synaptic and total gamma-aminobutyric acid A receptors (GABA_A_Rs) in the neuropil and in the plasma membrane of motoneurons. However, the effect of TNF-*α* on GABA_A_R trafficking was complex, displaying a nonlinear dose-dependent relationship [129]. The authors suggests that under certain physiological conditions GABAergic synapses can be excitatory and that excitatory effects of GABA_A_Rs have been implicated in maladaptive spinal plasticity in a model of instrumental learning [130]. Interestingly, the same group has also reported that TNF-*α* is necessary and sufficient for generating lasting inhibition of spinal learning and that the effect of this cytokine also involves Ca^2+^ permeable-AMPARs, since it was reverted by a GluR2-lacking AMPA receptor antagonist [131]. More recently, an* in vitro *study in mature rat and mouse hippocampal neurons in culture demonstrated that acute (45 min) application of TNF-*α* induced a rapid and persistent decrease of inhibitory synaptic strength as well as a downregulation of cell-surface levels of GABA_A_Rs. The trafficking of these receptors in response to TNF-*α* was mediated through the activation of neuronally expressed TNFR1 and required the activation of PI3-K, p38 MAPK, protein phosphatase 1, and dynamin GTPase [132].

Together, the findings presented here indicate that TNF-*α* potentiates excitotoxicity by rapidly increasing excitatory synaptic strength through increased AMPA and NMDA receptors surface expression and also that neurons respond to elevated levels of the cytokine weakening their inhibitory synaptic strength through a decreased presence of GABA_A_Rs in the plasma membrane. Thus, the net effect of TNF-*α* is to alter the balance of excitation and inhibition resulting in a higher synaptic excitatory/inhibitory ratio [111] (Figure 1). Interestingly, it has been proposed that an elevation of this ratio is a major cause of autism spectrum disorder [133, 134]; a pathology in that elevated levels of TNF-*α* in cerebrospinal fluid has been described [135].

## 7. TNF-*****α***** Links Neuroinflammation and Excitotoxicity

It is now widely accepted that most developmental, lesional, and degenerative nervous system disorders involve common interconnected neurotoxic mechanisms. Figure 1 summarizes the proposed mechanisms by which the cytokine TNF-*α* links the neuroinflammatory response to glutamate-mediated toxicity. The scheme can also be regarded as three interrelated vicious circles. The first is a microglial vicious circle in which TNF-*α* stimulates its own release. Then, it also stimulates glutamate release that acts on microglial metabotropic glutamate receptors to stimulate more TNF-*α* release. The second is an astroglial vicious circle in which TNF-*α* stimulates astrocytes to release glutamate that cannot be efficiently taken up by their glutamate transporters, thus increasing more and more the extracellular glutamate concentrations. The third is a neuronal vicious circle in which TNF-*α*, by increasing the synaptic excitatory/inhibitory ratio, induces an excessive calcium entry that results in excitotoxic neuronal death; the dying neurons keep microglia in an active state that maintains their increased TNF-*α* production and release. As TNF-*α* is released by activated microglia these mechanistic links between neuroinflammation and excitotoxicity can be considered as a crosstalk between microglia and astrocytes (modulating astrocytic glutamate uptake) and microglia and neurons (modulating neuronal glutamate and GABA receptors).

It is noteworthy that the scheme shown in Figure 1 not only accounts for the most common acute or chronic neurodegenerative diseases in which increased levels of TNF-*α*, associated with neuroinflammation and excitotoxicity, have been reported, but also describes a broader situation in which activated microglia releases significant amounts of TNF-*α*. This is the case of opioid tolerance and neuropathic pain, two situations that are modulated by TNF-*α* [136, 137]. Chronic morphine exposure induces microglial activation and a significant increase in TNF-*α* mRNA expression in the rat spinal cord [138]; this effect is associated with a downregulation of GLT-1 and GLAST glutamate transporters and with an increase in the surface expression of Ca^2+^ permeable-AMPA and NMDA receptors [139]. All the above effects of chronic morphine, and remarkably, the loss of its antinociceptive effect, can be reverted by a TNF-*α* antagonist [138, 139]. Similarly, in mechanical allodynia, TNF-*α* mediated increased insertion of Ca^2+^ permeable-AMPARs in spinal cord neurons plays a major role in inflammatory pain and may represent a path by which glia contribute to neuronal sensitization and pathological pain [140].

## 8. Therapy Targeting TNF-*****α*****

As TNF-*α* is a key mediator in the pathological mechanisms of a large number of neurological disorders including ischemia, AD, PD, MS, and ALS [3] and also in peripheral autoimmune disorders including rheumatoid and juvenile arthritis, ankylosing spondylitis, and Crohn's disease, targeting TNF-*α* action seems to be an attractive disease-modifying strategy. The different strategies employed for TNF-*α* inhibition have been reviewed elsewhere [12] and include the use of humanized IgG antibodies (infliximab, adalimumab, and etanercept) that sequestrate sTNF-*α* and tmTNF-*α*, the antibiotic minocycline that decreases TNF-*α* synthesis, the immunomodulatory drug thalidomide and its derivatives that enhance the degradation of TNF-*α* mRNA [13], and TACE inhibitors that inhibit sTNF-*α* production. Clinical trials examining the effects of TNF-*α* inhibition have been conducted on patients with MS, AD, and ALS. Although promising effects were obtained in AD patients with substantial cognitive and behavioral improvements [141, 142], the treatment failed in MS and ALS patients [143, 144]. Moreover, TNF-*α* gene knockout did not affect life span or the extent of motoneuron loss in the superoxide dismutase 1 (SOD1) transgenic mice model of ALS, thus suggesting that TNF-*α* alone is not a key factor in motoneuron degeneration [145].

The above findings can be explained first because TNF-*α* has both neuroprotective and neurotoxic effects related to the different signaling pathways activated by their receptors [146]. In this sense, mice lacking TNF-*α* receptors were more susceptible to ischemia and excitotoxic injury [147, 148]. Second, because some proinflammatory cytokines (i.e., IL-1*β* and TNF-*α*) have redundant functions* in vivo*; thus, in the TNF-*α* knockout mice an increase in the transcripts encoding for IL-1*β* was detected [145]; and, third, because TNF-*α* often works in concert with other cytokines (i.e., IFN-*γ* and IL-1*β*) to promote neuronal death [66, 149, 150]. Nevertheless, the identification of novel agents that can restore the normal function of activated glial cells by means of reducing the production of TNF-*α* and/or its potentiation of excitotoxicity will be essential in the management of chronic and acute neurodegenerative diseases.

## 9. Conclusion

TNF-*α* plays a physiological role in controlling synaptic transmission and plasticity in the healthy CNS by modulating ionotropic glutamate receptors trafficking. However, excessive TNF-*α* levels, as a result of different types of injury, have an inhibitory effect on glutamate transporters, resulting in increased glutamate concentration in the CNS parenchyma. In this context, even slight increases in TNF-*α* induced Ca^2+^ permeable-AMPA and/or NMDA receptors trafficking become toxic for neurons. As microglial activation and upregulation of TNF-*α* expression is a common feature of several CNS diseases, as well as chronic opioid exposure and neuropathic pain, modulating TNF-*α* signaling may represent a valuable target for intervention.

## Abbreviations

A-SMase: Acidic sphingomyelinase
AD: Alzheimer's disease
ALS: Amyotrophic lateral sclerosis
AMPA: *α*-Amino-3-hydroxy-5-methyl-4-isoxazolepropionic
AMPAR: AMPA-type glutamate receptor
CNS: Central nervous system
EAAT: Excitatory amino acid transporter
ERK: Extracellular signal-regulated kinase
GABA_A_R: Gamma-aminobutyric acid A receptor
GLAST: Glutamate/aspartate transporter
GLT-1: Glutamate transporter 1
GluR: Glutamate receptor subunit
IFN-*γ*: Interferon gamma
IFN-*γ*R: Interferon gamma receptor
JNK: c-Jun N-terminal kinase
KA: Kainate
MEK: Mitogen-activated protein kinase and ERK kinase
mGluR2: Group 2 metabotropic glutamate receptor
MHC: Major histocompatibility complex
MS: Multiple sclerosis
N-SMase: Neutral sphingomyelinase
NF-*κ*B: Nuclear factor-kappa B
NMDAR: N-methyl-D-aspartate receptor
p38 MAPK: p38 mitogen-activated protein kinase
PD: Parkinson's disease
PI3-K: Phosphatidylinositol 3-kinase
ROS: Reactive oxygen species
sTNF-*α*: Soluble TNF-*α*
THA: Threohydroxyaspartate
tmTNF-*α*: Transmembrane TNF-*α*
TNF-*α*: Tumor necrosis factor alpha
TNFR1: TNF-*α* receptor 1
TNRF2: TNF-*α* receptor 2.
